# Dysfunctional mitochondria accumulate in a skeletal muscle knockout model of *Smn1*, the causal gene of spinal muscular atrophy

**DOI:** 10.1038/s41419-023-05573-x

**Published:** 2023-02-27

**Authors:** Francesco Chemello, Michela Pozzobon, Lorenza Iolanda Tsansizi, Tatiana Varanita, Rubèn Quintana-Cabrera, Daniele Bonesso, Martina Piccoli, Gerolamo Lanfranchi, Marta Giacomello, Luca Scorrano, Camilla Bean

**Affiliations:** 1 Department of Biology, University of Padova, 35121 Padova, Italy; 2 Women’s and Children’s Health Department, University of Padova, 35121 Padova, Italy; 3 Foundation Institute of Pediatric Research Città della Speranza, 35129 Padova, Italy; 4 Veneto Institute of Molecular Medicine, 35129 Padova, Italy; 5 Department of Medicine, University of Udine, 33100 Udine, Italy; 6 Present Address: Department of Molecular, Cellular and Developmental Neurobiology, Cajal Institute, CSIC, Madrid, Spain

**Keywords:** Transcriptomics, Gene expression profiling

## Abstract

The approved gene therapies for spinal muscular atrophy (SMA), caused by loss of survival motor neuron 1 (SMN1), greatly ameliorate SMA natural history but are not curative. These therapies primarily target motor neurons, but SMN1 loss has detrimental effects beyond motor neurons and especially in muscle. Here we show that SMN loss in mouse skeletal muscle leads to accumulation of dysfunctional mitochondria. Expression profiling of single myofibers from a muscle specific *Smn1* knockout mouse model revealed down-regulation of mitochondrial and lysosomal genes. Albeit levels of proteins that mark mitochondria for mitophagy were increased, morphologically deranged mitochondria with impaired complex I and IV activity and respiration and that produced excess reactive oxygen species accumulated in *Smn1* knockout muscles, because of the lysosomal dysfunction highlighted by the transcriptional profiling. Amniotic fluid stem cells transplantation that corrects the SMN knockout mouse myopathic phenotype restored mitochondrial morphology and expression of mitochondrial genes. Thus, targeting muscle mitochondrial dysfunction in SMA may complement the current gene therapy.

## Introduction

Spinal muscular atrophy (SMA) is a severe motoneuron disease caused by a genetic defect in the *SMN1* gene encoding for the survival motoneuron protein SMN1. The loss of SMN1 is lethal in human. The motor neurons of the anterior horns of the spinal cord degenerate in SMA patients, resulting in fatigue, atrophy of the proximal muscles, and paralysis of respiratory muscles. In most tissues, a paralogue human gene (*SMN2*) exists. However, this gene is aberrantly spliced originating an unstable, degraded SMN protein. Only 10–20% of *SMN2* mRNA originates a full-length stable SMN protein that can rescue SMN1 loss. The existence of *SMN2* constitutes the rationale for two of the three recently approved gene treatments for SMA [1]. While all these 3 therapies aim at SMN restoration, Nusinersen and Risdiplam target SMN2, whereas Zolgensma targets SMN1. Nusinersen is an AntiSense Oligonucleotide (ASO) that binds to a specific sequence in the *SMN2* RNA altering its splicing pattern to produce a functional SMN protein only in the central nervous system; Risdiplam works by correcting SMN2 splicing preventing the removal of exon 7 from the mature mRNA; Zolgensma is an adeno-associated viral vector (AAV) gene therapy that delivers the SMN transgene and produces the full-length functional SMN protein. While treated patients show improvements in neuromotor tone and lifespan, ongoing loss of SMN function and presence of neuromuscular symptoms remain. In fact, patients still exhibit motor function deficits [2, 3] and the muscle atrophy that characterizes SMA cannot be rescued [4]. Although SMA has long been considered a disease restricted to motor neurons, the involvement of other tissues and cells including skeletal muscle [5–8], pancreas [9, 10], liver [9–11], kidney [12], spleen [13, 14], vasculature [15, 16], heart [17–19] and Schwann cells [20–22] is now clear.

Because of the ubiquitous expression of SMN and especially in the skeletal muscle, drugs that target skeletal muscle may enhance the efficacy of the therapies upregulating SMN and slowing the central process of motor neuron loss. In fact, novel muscle-specific therapies that inhibit myostatin (SRK-015) [23] or sensitize the troponin complex to lower the threshold for muscle contractility and to reduce the metabolic energy cost of contraction (CK-2127107) [24] are currently in clinical trial. Promising early results show that skeletal muscle could be targeted to improve motor function in SMA.

A better understanding of the molecular mechanisms underlying the unique role of skeletal muscle in SMA disease may help the design of rationale therapies. With this question in mind, we set out to investigate the pathobiology of SMN loss in a mouse model of conditional skeletal muscle *Smn1* deletion. By combining single fibers transcriptional analyses and functional studies, we show that SMA muscles accumulate dysfunctional mitochondria that are not selectively removed. These altered mitochondria impair Ca^2+^ buffering and lead to the accumulation of reactive oxygen species (ROS). Transplantation of amniotic fluid stem (AFS) cells that have been shown to ameliorate the phenotype of a muscle-specific SMA model [25] also recovers mitochondrial function in the *Smn1* deleted mouse model. Our findings point to the mitochondria of skeletal muscle as therapeutic targets additional to *SMN1* and *SMN2* in SMA.

## Materials and methods

### Mice

Male C57BL/6 (Ly5.2) GFP^+/−^ mice and female C57BL/6 (Ly5.2) GFP^−/−^mice were used to obtain both Ly5.2 GFP^+/−^and GFP^−/−^embryos. *HSA‐Cre, Smn*^*F7/F7*^mice (from now on *Smn1*^*ΔSkm*^*)* were used as recipients. Male mice were used for the experiments. Sample size was chosen to use the fewest number of animals to achieve statistical significance; no statistical methods were used to predetermine sample size. Mouse experiments were not randomized since the mice used for the study were identified by genotyping. In other experiments, when mice were treated they were randomly assigned to no treatment or AFS cells treatment. Blinding was not always possible during data collection because mouse phenotypes were easily linkable to the corresponding genotypes. The investigators were blinded to group allocation during data analyses. All experimental samples were included in the analyses, with no data excluded. All procedures were approved by the University of Padua’s Animal Care and Use Committee (CEASA, protocol references 107/07 and 41056) and, in accordance with Italian law, were communicated to the Ministry of Health and local authorities.

### Amniotic fluid collection and stem cell selection

Amniotic fluid samples have been collected as already described [25]. Briefly, murine amniotic fluid was extracted from fetuses (E11.5–13.5) and c‐kit^+^ cells were isolated using the Miltenyi Mouse Lineage Cell depletion kit and then CD117 MicroBeads (all from Miltenyi Biotech, Cologne, Germany). The cells freshly isolated have been already characterized [26].

### Cell injection

Three‐month‐old *Smn1*^*ΔSkm*^ mice were randomized to receive either no treatment or AFS cells as described in Piccoli et al. [25]. In vitro expanded AFS cells were sorted with an Aria FACS system (Becton Dickinson, Franklin Lakes, New Jersey, USA), selected for the expression of GFP and c‐kit and injected via tail vein in *Smn1*^*ΔSkm*^ mice. One month after post-transplantation mice were sacrificed to isolate single muscle fibers.

### Single fiber isolation

Single myofibers were isolated from AFS-transplanted animals and from C57BL/6 GFP^+^ mice, as previously described [25]. Briefly, *tibialis anterior* muscles were dissociated 2 h at 37 °C in 0.2% (wt/vol) type I collagenase (Sigma-Aldrich, St.Louis, Missouri, USA). Following digestion, dissociated muscles were transferred in DMEM (low glucose; Invitrogen, Waltham, Massachusetts, USA) on a horse serum (Invitrogen)-coated 10 cm dish (Falcon; BD Biosciences) and gently triturated with a wide-bore pipette to release single myofibers. Myofibers were individually collected under a stereo microscope.

### Microarray profiles

Amplification and labeling of RNA from single fibers were fully described in our previous work [27]. Briefly, RNA was purified from each single myofiber and exponentially amplified using the TransPlex Whole Transcriptome Amplification 2 Kit (Sigma-Aldrich). RNA was reverse-transcribed in a cDNA library, and the library was amplified for 18 cycles. cDNA was then labeled by using the Genomic DNA Enzymatic Labeling Kit (Agilent Technologies, Santa Clara, CA, USA). Labeled cDNA was purified using the Amicon 30 kDa filters (Millipore, Burlington, MA) and quantified. Microarray experiments were performed as previously described [27] with SurePrint G3 Mouse Gene Expression 8 x 60 K microarray platforms (Agilent Technologies).

### Microarray data processing and statistical analyses

All the procedures were already described [27]. Briefly, probes features were extracted. Intra- and inter-array normalizations were directly performed, and data were Log2 transformed and not available (NA) value was assigned if Found and/or Well Above Background flags were not positive. Expression values of probes with the same sequence were mediated. Only probes with at least 75% available values (i.e. no more than 25% of NA values) were taken into consideration for the further analyses.

In myofiber microarray experiments, 8, 12 and 7 myofibers isolated from respectively Wt, *Smn1*^*ΔSkm*^ and AFS-treated *Smn1*^*ΔSkm*^ mice (total: 25 microarrays) were analyzed.

Microarray data were analyzed using the MultiExperiment Viewer (MeV, Ver. 4.8), a tool of TM4 Microarray Software Suite [28].

Samples (myofibers) were hierarchically clustered using the Pearson’s correlation distance and average linkage method. To identify the differentially expressed (DE) genes, we performed unpaired two-class Significance Analysis of Microarrays (SAM) [29]. Genes with a q-value <0.001 (based on 1000 permutations) were considered as DE.

DE genes were categorized in gene ontology (GO) classes using the Functional Annotation Clustering method, a bioinformatic tool of the DAVID database [30]. Only GO terms with an enrichment score greater than 1.3 and with *P* value less than 0.05 were considered.

Genes that significantly correlate with a defined template expression pattern were identified by using the Pavlidis Template Matching algorithm [31] in TIGR Multi Experiment Viewer.

The oPOSSUM tool was used to detect the enrichment of transcription factors in the promoter of DE genes [32].

### Microarray data availability

The accession number for the raw microarray data reported in this paper is NCBI GEO: GSE207890.

### Transmission EM

Skeletal muscle specimens from Wt and *Smn1*^*ΔSkm*^ mice were fixed in 2% formaldehyde, 2.5% (V/V) glutaraldehyde in 0.1 M Na-cacodylate (pH 7.4) overnight at 4 °C. EMs were acquired as described [27]. Quantification of mitochondrial morphometry (area, perimeter, total number of damaged mitochondria) in electron micrographs was performed using ImageJ.

### Western blot analysis

Skeletal muscle mouse tissues were homogenized and lysed in RIPA buffer supplemented with protease inhibitors (PIC, Sigma-Aldrich). Protein concentration was determined using Pierce BCA Protein Assay Kit (Thermo Fisher Scientific, Waltham, MA, USA). Lysates were then boiled in sample buffer (Invitrogen) supplemented with 1 mM dithiothreitol, separated on gels, and transferred to polyvinylidene difluoride (PVDF) membranes (Immobilon P, Millipore). Membranes were probed with the following antibodies: NDUFA9 (Abcam, Cambridge, UK, ab14713); SDHA (Abcam, ab14715); ATPV5 (Abcam, ab14748); TOMM20 (Thermo Fisher Scientific, MA524859); TUBULIN (Santa Cruz Biotechnology, USA sc-5286); SMN1 (Santa Cruz Biotechnology, sc-15320); GADPH (Sigma-Aldrich, G9545); p62 (MBL International, USA, PM045); PINK (Novus Biologicals, BC100-494); VINCULIN (Merck V9264); LC3 (MBL International, PM036); PARKIN (Santa Cruz Biotechnology sc-32383); LAMP1 (Abcam, ab25245); LAMP2 (Abcam, ab203224); eiF2a (Cell Signaling Technology, 5324) and P-eiF2a (Cell Signaling Technology, 3398).

### Measurement of oxygen consumption

Mitochondria were isolated by differential centrifugation from skeletal muscles of Wt and *Smn1*^*ΔSkm*^ mice as previously described [33]. For oxygraphic measurements, 250–500 μg of mitochondrial protein extracts were incubated in a buffer containing 225 mM sucrose, 75 mM mannitol, 10 mM Tris-HCl (pH 7.4), 10 mM KCl, 10 mM KH_2_PO_4_, 5 mM MgCl_2_, and 1 mg/ml fatty-acids-free BSA (pH 7.4). Oxygen consumption was evaluated by a Clark oxygen electrode (Hansatech, Instruments). After OCR baseline was established, a solution containing 5 mM succinate and 2 μM rotenone for cII-dependent respiration, ADP to stimulate ATP-coupled oxygen consumption, oligomycin and FCCP were sequentially added.

### Blue native gel electrophoresis

Blue native gel electrophoresis (BNGE) analysis was performed as described in [33]. Briefly, 250 μg of isolated muscle mitochondria were resuspended in native page buffer (Invitrogen), protease inhibitors, and 4% digitonin, incubated for 1 h on ice and centrifuged at 20,000 × *g* at 4 °C. after adding 5% Coomassie G250. 30 μg were separated by 3–12% gradient BNGE and stained with for in-gel activities.

### Biochemical analysis of MRC complexes

Skeletal muscle mitochondria were resuspended in 10 mM Tris-HCL pH 7.6. The spectrophotometric activity of complex I (CI), complex II (CII), complex III (CIII), and complex IV (CIV), as well as citrate synthase (CS), was measured as described in Brischigliaro et al. [34].

### Cytochrome c oxidase assay

Transverse sections (10 μm thick) of isopentane‐frozen *gastrocnemius* muscle of 3 months old Wt and *Smn1*^*ΔSkm*^ mice were incubated at 22 °C for 30 min with PMS (0.2 mm), NBT (2 mm), and sodium succinate (130 mm) in the absence of any COX inhibitors [35]. Sections were mounted with Eukitt mounting medium (BIOSIGMA, Italy), observed under Leica DM6 microscope (Leica), and pictures taken using Leica LAS X Suite software.

### Measurement of H_2_O_2_ release in permeabilized fibers

Dissection and permeabilization of fibers bundles with saponin were performed as described previously [36]. Briefly, *tibialis anterior* muscles were removed from Wt and *Smn1*^*ΔSkm*^ mice and placed into precooled buffer X (2.77 mM CaK_2_EGTA, 7.23 mM K_2_EGTA, 6.56 mM MgCl_2_, 0.5 mM dithiothreitol (DTT), 50 mM K-MES, 20 mM imidazol, 20 mM taurine, 5.3 mM Na_2_ATP, 15 mM phosphocreatine, pH 7.3 at 4 °C) containing saponin (30 µg/ml). After 30 min of incubation with mild shaking, 3 washes with buffer Z containing 30 mM KCl, 10 mM KH_2_PO_4_, 5 mM MgCl_2_-6H_2_O, 105 mM K-MES, and 0.5 mg/ml BSA were performed. H_2_O_2_ emission was measured in permeabilized fiber bundles by using Amplex Red Hydrogen Peroxide/Peroxidase Assay Kit (Thermo-Fisher). The fluorescence signal was then detected in a plate reader (Fluoroskan, Thermo Scientific) with excitation at 544 nm and emission at 590 nm.

### Cytosolic and mitochondrial calcium determination

Single muscle fibers were isolated from *flexor digitorum brevis* (FDB) muscle from mice of the indicated genotypes as previously described [27]. Isolated fibers were left to sediment onto a Matrigel®-coated 35 mm-dish overnight (BD Biosciences). Fibers were then loaded with Fura2-AM (5 µM, Molecular Probes, USA), pluronic acid 0.04% and sulfinpyrazione 250 µM, at 37 °C for 40 min, to measure cytosolic calcium transients. Fibers were instead loaded with Rhod 2AM (5 µM, Molecular Probes) and Hoechst 33342 (0,2 µg/ml) for 30 min at 37 °C and 15 min at room temperature. Fura2 signal was acquired in single fibers at rest and after administration of 30 mM caffeine (Sigma).

### Tetramethylrhodamine, methyl ester (TMRM) assay

Single muscle fibers were isolated from *tibialis anterior* (TA) muscles from mice of the indicated genotypes as previously described [27], and loaded with 20 nM tetramethyl rhodamine methyl ester (TMRM) in media supplemented with glucose and cyclosporin H (1 µM, Merck) to inhibit the plasma membrane multidrug resistance pump), for 30 min at 37 °C. TMRM fluorescence intensity was measured using an Operetta (Perkin Elmer) high content imaging system every 15 min. Where indicated, oligomycin (5 µM, Merck) or 10 µM FCCP were added.

### Cell culture and treatments and lentiviral transduction

C2C12 (ATCC, CRL-1772) myoblasts were grown and led to differentiate into myotubes as previously described [27]. Cells tested negative for mycoplasma in routine, monthly testing. C2C12 myoblasts were seeded at 80% confluency and transfected using Lipofectamine 2000 (ThermoFisher Scientific) following the manufacturer’s instructions with 75 nM of Silencer Select siRNA for *Smn1* (4390816) or Silencer Select scrambled sequence, purchased from ThermoFisher Scientific.

Knockdown of *Smn1* in C2C12 differentiated myotubes for 10 days was performed by lentiviral delivery of shRNA. Viral particles produced from four individual MISSION shRNA were used to find the highest level of knockdown (Sigma-Aldrich TRCN0000072019, TRCN0000072020, TRCN0000072021, TRCN0000072022); non-target shRNA (Sigma-Aldrich SHC016) was used as control. Myoblasts and myotubes were collected for electron microscopy analyses two days after transfection or transduction.

Autophagic flux was performed in C2C12 myoblasts transfected with siScr or siSmn1. After 2 days from transfection C2C12 were treated for two hours before collection with 200μM chloroquine (CQ, C6628 Sigma Aldrich).

### Statistical analyses

Data are presented as mean ± s.e.m. In dot-box plots, dots represent the individual measurements, boxes represent the mean ± s.e.m. and whiskers the 10th and 90th percentiles. Independent experiments were performed and the specific number of biological replicates are indicated in the figure legends. Sample size was predetermined based on published literature and previous lab experience. The investigators were blinded to group allocation during data analysis. No data were excluded from the analyses. Normal distribution of data was verified by a Shapiro–Wilkinson test. If data were not normally distributed, the non-parametric Mann–Whitney *U*-test was used to evaluate significance; otherwise, the appropriate parametric *t*-test was used, as specified in the figure legends.

## Results

### Gene expression analyses at the single muscle fiber level reveal the impairment of mitochondria related processes in *Smn1* deficient muscle

**Fig. 1:**
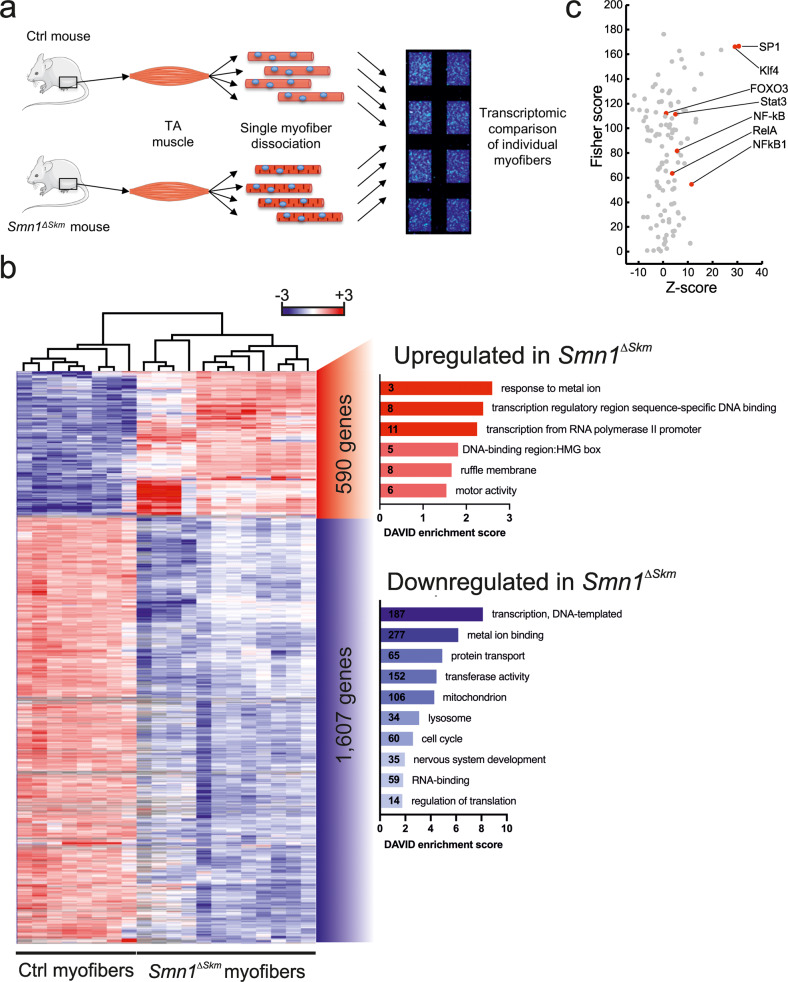
Single myofiber gene signatures reveal the transcriptional changes induced by SMN loss in skeletal muscle. **a** Schematic representation of the experimental steps leading to microarrays of cDNA produced from RNA extracted from single myofibers of the indicated genotypes. **b** Cluster analysis of the differentially expressed genes (DEGs) distinguishes the signatures of myofibers isolated from *tibialis anterior* muscles of Wt and *Smn1*^*ΔSkm*^ mice. Gene ontology (GO) analysis of over-and under-expressed genes in response to SMN loss. Enrichment score by DAVID was used to rank the annotation groups. The numbers inside bars correspond to the number of DEGs in each cluster. **c** Plot of the Fisher score (y-axis) vs. the Z-score (x-axis) from the oPOSSUM transcription factor enrichment analysis of the list of DEGs in **b**. Each transcription factor is represented by a dot on the graph; transcription factors whose binding sites are most strongly enriched within the promoters of the specified gene list have high scores (top right of plot).

Several studies highlighted the involvement of tissues other than motor neurons in the pathophysiology of SMA, including skeletal muscle. Here we wished to determine in an unbiased fashion which cellular and molecular pathways link marked muscle-specific deficiency of *Smn1* to SMA pathology. To this end we capitalized on a model of selective skeletal muscle *Smn1* knockout [37] (HSA-Cre:Smn^F7/F7^, from now on *Smn1*^*ΔSkm*^). Our genome-wide transcriptomic approach based on single myofiber gene expression profiling (microgenomics [38]) distinguishes alterations of individual muscle fibers from those occurring in non-muscle cells (Fig. 1a). Twelve myofibers isolated from *tibialis anterior* (TA) muscle of three months old muscle specific *Smn1*^*ΔSkm*^ and eight myofibers from TA of control littermates were independently profiled. The diversity between mutated and wild type (Wt) myofibers could be unambiguously recognized at transcriptional level, since all *Smn1*^*ΔSkm*^ myofibers clustered in one distinct group well separated from the control myofibers (Fig. 1b). We identified 2197 non-redundant differentially expressed (DE) genes between single mutated *Smn1*^*ΔSkm*^ and control myofibers: in total 590 genes were found over-expressed in *Smn1*^*ΔSkm*^ and 1607 in control myofibers (Fig. 1b, Supplementary Table 1). The signatures produced with this approach are much richer in muscle-specific information, so the transcriptional changes reflected the real effects of the loss of *Smn1* function in skeletal muscle. Gene Ontology (GO) enrichment analysis in *Smn1*^*ΔSkm*^ myofibers detected six GO terms over-represented in the list of overexpressed genes and twelve terms in the list of downregulated genes (Fig. 1b). Notably, many genes with regulatory functions were found by GO analysis under the categories ‘transcription and translation regulation’ and ‘RNA binding’. The SMN depletion evoked an immediate reprogramming of gene expression in myofibers. We investigated which regulators were associated with the transcriptional reprogramming in myofibers upon SMN depletion. We looked at the over-represented transcription factor binding sites in the promoters of the DEGs. Interestingly, recognition sites of critical regulators of autophagy/mitophagy such as NF-kB and FOXO transcription factors [39, 40] were found enriched (Fig. 1c).

**Fig. 2:**
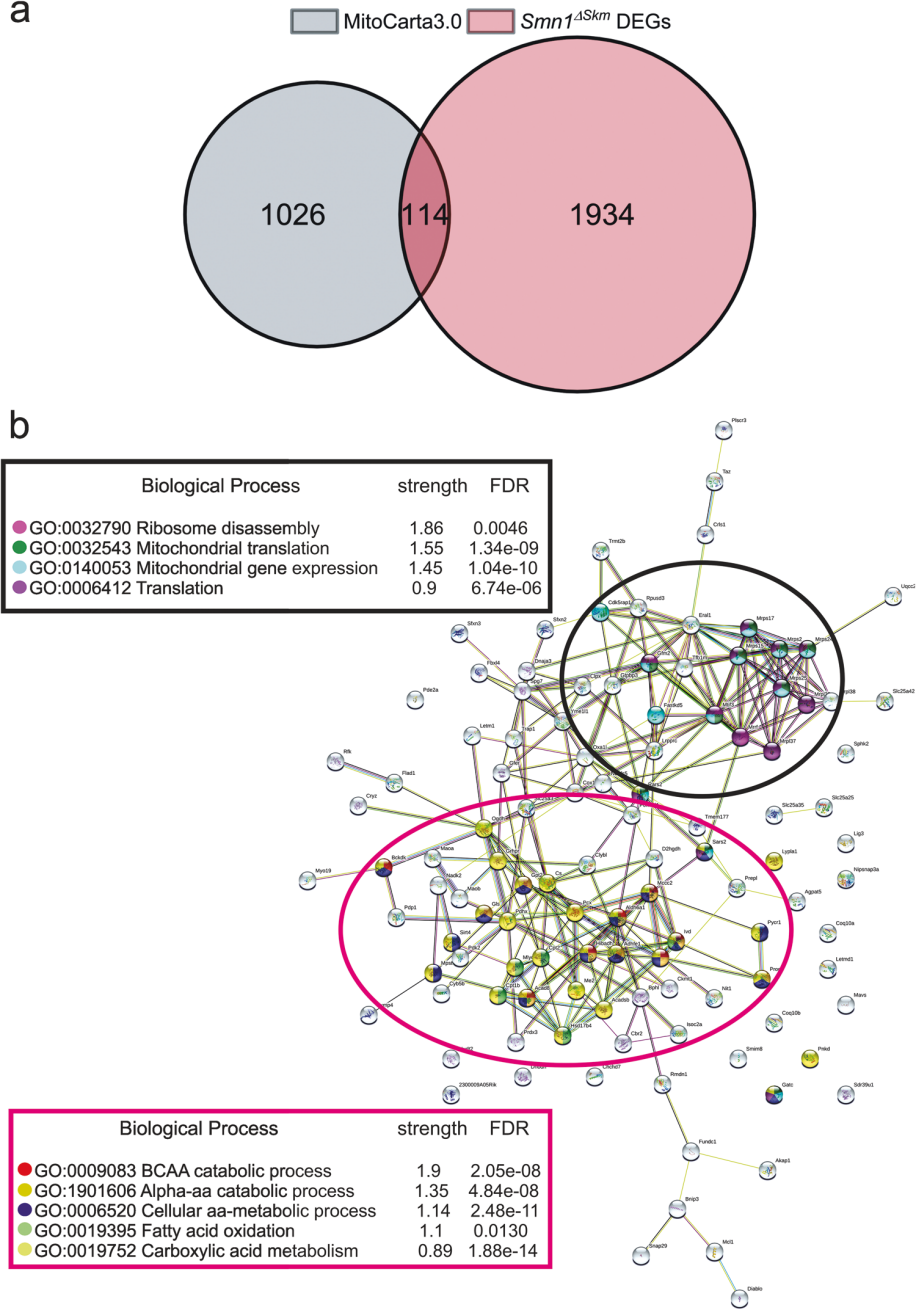
SMN loss leads to global changes of mitochondrial genes transcription. **a** The 2197 DEGs in *Smn1*^*ΔSkm*^ myofibers were cross-referenced against MitoCarta 3.0 to identify those that have mitochondrial localization. **b** The 114 mitochondrial DEGs in **a**) were mapped to the STRING database to find the potential protein-protein interactions (PPI). The PPI network identified 2 main functional modules enriched by integrating GO function analyses.

Consistently, the functional annotation drew special attention to processes associated to mitochondrial biology and lysosomal function. It is well known that dysfunctional mitochondria and lysosome are associated with neurological diseases [41, 42], suggesting that impairments of mitochondria and lysosomes could contribute to SMA pathogenesis too. Notably, 10% of the known mouse mitochondrial genes annotated in the MitoCarta 3.0 collection [43] were found differentially expressed in *Smn1*^*ΔSkm*^ myofibers (Fig. 2a; Supplementary Table 2). Protein-protein interaction (PPI) network analysis of this set of genes by STRING [44] revealed the significant functional clusters in which the major biological processes were involved in mRNA and amino acid metabolism and in particular the essential branched-chain amino acids (Fig. 2b). Our data suggest that SMN loss in skeletal muscle could affect key processes for the maintenance of mitochondrial health.

### Severe ultrastructural and functional alterations in the mitochondria of *Smn1*^*ΔSkm*^ myofibers

The transcriptional changes observed in *Smn1*^*ΔSkm*^ fibers prompted us to check whether mitochondrial morphology and function were affected. We performed transmission electron microscopy (EM) on transverse sections of TA muscles of Wt and *Smn1*^*ΔSkm*^ mice.

**Fig. 3:**
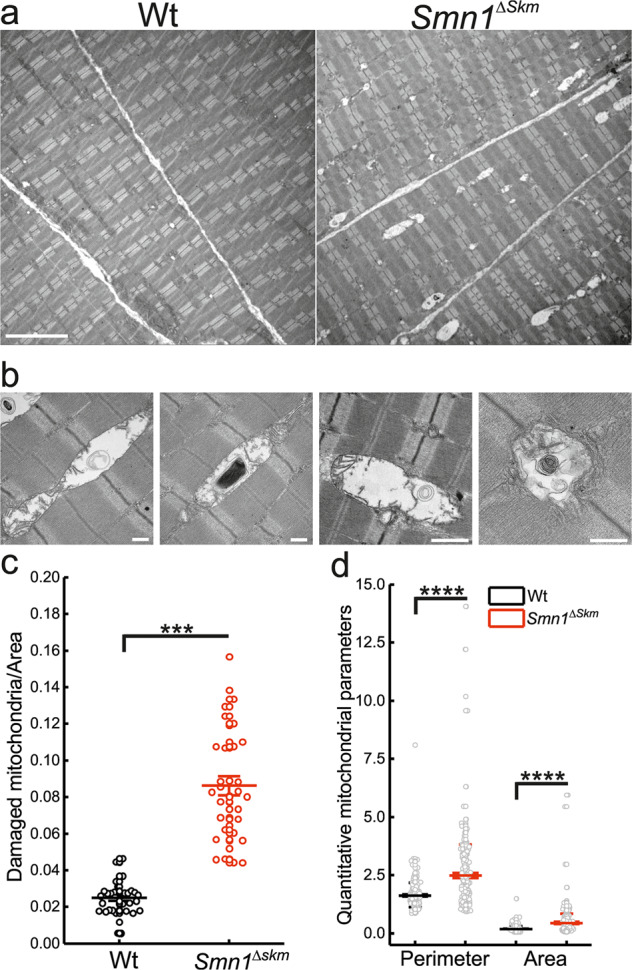
Skeletal muscle mitochondria of *Smn1*^*ΔSkm*^ mice show ultrastructural abnormalities. **a** Representative electron micrographs of mitochondria from TA muscles of the indicated genotypes. Scale bar: 5 μm. **b** Representative high magnification of abnormal mitochondria in TA muscle of *Smn1*^*ΔSkm*^ mice. Scale bar: 0.5 μm. **c** Box-dots plots of the number of damaged mitochondria per area of Wt and *Smn1*^*ΔSkm*^ TA muscles. The error bars indicate SEM; ***p = 2.8E-12. **d** Morphometric quantification of the area and perimeter of mitochondria from the different genotypes. >200 mitochondria from Wt and *Smn1*^*ΔSkm*^ mice (*n* = 3) were analyzed. The error bars indicate SEM; ****p ≤ 10E-17.

EM images revealed that mitochondria of *Smn1*^*ΔSkm*^ muscle exhibited numerous and heterogeneous ultrastructural alterations. In control TA muscle, mitochondrial doublets were typically round and encircled myofibers at the I-bands, where they coupled to Ca^2+^ release units (triads) similarly to what had been previously shown in a healthy muscle [45]. Oppositely, many mitochondria in TA fibers from *Smn1*^*ΔSkm*^ mice appeared abnormal and damaged (Fig. 3a).

The abnormal mitochondria in *Smn1*^*ΔSkm*^ TA fibers were swollen and variable in size. The internal cristae appeared disorganized and disrupted or, at times, completely missing: some mitochondria displayed an almost completely clear matrix and others contained electron-dense deposits (vacuoles or lamellar structures) that were associated with ruptured cristae (Fig. 3b). Fibers from *Smn1*^*ΔSkm*^ mice presented with 3-fold more damaged mitochondria than controls (Fig. 3c). A detailed morphometric analysis revealed that the perimeter and area of the *Smn1*^*ΔSkm*^ TA fibers mitochondria were significantly increased compared to fibers from their Wt littermates (Fig. 3d).

**Fig. 4:**
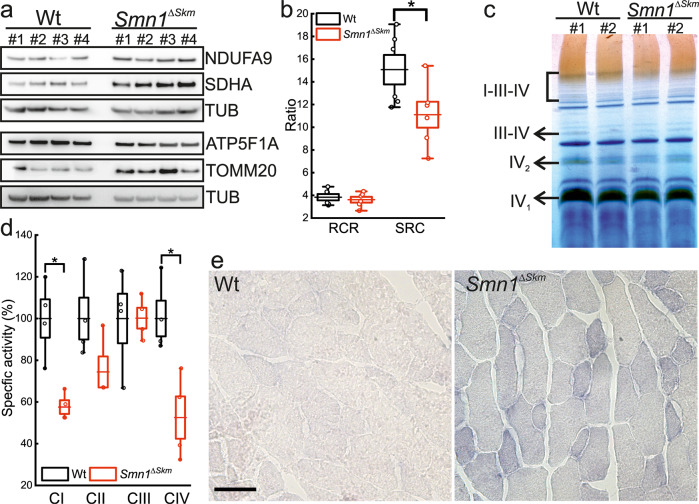
Skeletal muscle mitochondria of *Smn1*^*ΔSkm*^ mice show altered functionality. **a** Equal amounts of TA muscle lysates (20 µg) were separated by SDS-PAGE and immunoblotted using the indicated antibodies. Each lane corresponds to an individual mouse of the indicated genotypes. Densitometry analysis yielded the following values: NUDFA9: 0.56 ± 0.05 in Wt (*n* = 4) and 0.9 ± 0.05 in *Smn1*^*ΔSkm*^ (*n* = 4), *p* = 0.03; SDHA: 0.28 ± 0.034 in Wt (*n* = 4) and 0.64 ± 0.036 in *Smn1*^*ΔSkm*^ (*n* = 4), *p* = 0.03; ATP5F1A: 1.09 ± 0.12 in Wt (*n* = 4) and 1.57 ± 0.08 in *Smn1*^*ΔSkm*^ (*n* = 4), *p* = 0.03; TOMM20: 0.5 ± 0.07 in Wt (*n* = 4) and 1.44 ± 0.26 in *Smn1*^*ΔSkm*^ (*n* = 4), *p* = 0.03. TUB is used as loading control. **b** Box-dots plots of respiratory control ratio (RCR) and spare respiratory capacity (SRC) measured in mitochondria isolated from skeletal muscles of Wt and *Smn1*^*ΔSkm*^ mice (*n* = 3). The error bars indicate SEM; *p = 0.045. **c** BNGE-in-gel activities of MRC CIV from BNGE of digitonin-treated isolated mitochondria from the indicated genotypes. **d** Mitochondria respiratory chain enzymatic activities normalized by citrate synthase activity in isolated muscle mitochondria from Wt and *Smn1*^*ΔSkm*^ muscle (*n* = 4). **e** Representative images of *gastrocnemius* muscle sections from mice of the indicated genotype stained with the NBTx method. Scale bar: 50 µm.

Functionally, in contrast to changes in the expression of key mitochondrial proteins (Fig. 4a; Supplementary Fig. 1a–d), there were no changes in the respiration of mitochondria purified from *Smn1*^*ΔSkm*^ muscles compared with Wt. However, while basal respiration condition was comparable, the spare respiratory capacity (SRC) was found severely reduced in mitochondria isolated from *Smn1*^*ΔSkm*^ muscles (Fig. 4b). Reduced SRC levels can correspond to mitochondrial dysfunction undetectable under basal conditions. SRC depends on multiple mitochondrial parameters, such as the quality and quantity of mitochondria, their metabolism and the activity and the assembly of respiratory chain components. Interestingly, complex IV enzymatic activity was reduced in *Smn1*^*ΔSkm*^ muscles, potentially explaining the observed drop in SRC levels (Fig. 4c; Supplementary Fig. 1e, f). Indeed, certain structural subunits and assembly factors of complex IV such as *Cox18*, *Tmem177* and *Oxa1l* that associate with complex IV defect [46, 47] were significantly downregulated in *Smn1*^*ΔSkm*^ myofibers (Supplementary Table 1). It has been reported that an assembled complex IV is required to maintain the stability of complex I [48, 49] and to form the I-III-IV supercomplex that was found reduced in *Smn1*^*ΔSkm*^ muscles (Fig. 4c). The spectrophotometric assay of mitochondrial respiratory chain enzyme activities revealed no significant changes in the activity of complexes II and III, but the activity measurements for complex IV and I were 50% decreased in *Smn1*^*ΔSkm*^ muscles compared to the controls (Fig. 4d).

Finally, a recently developed assay able to detect dysfunctional COX activity [35] unambiguously confirmed COX-deficiency in *Smn1*^*ΔSkm*^ myofibers (Fig. 4e).

### Increased ROS production and Ca^2+^ accumulation in *Smn1*^*ΔSkm*^ myofiber mitochondria

**Fig. 5:**
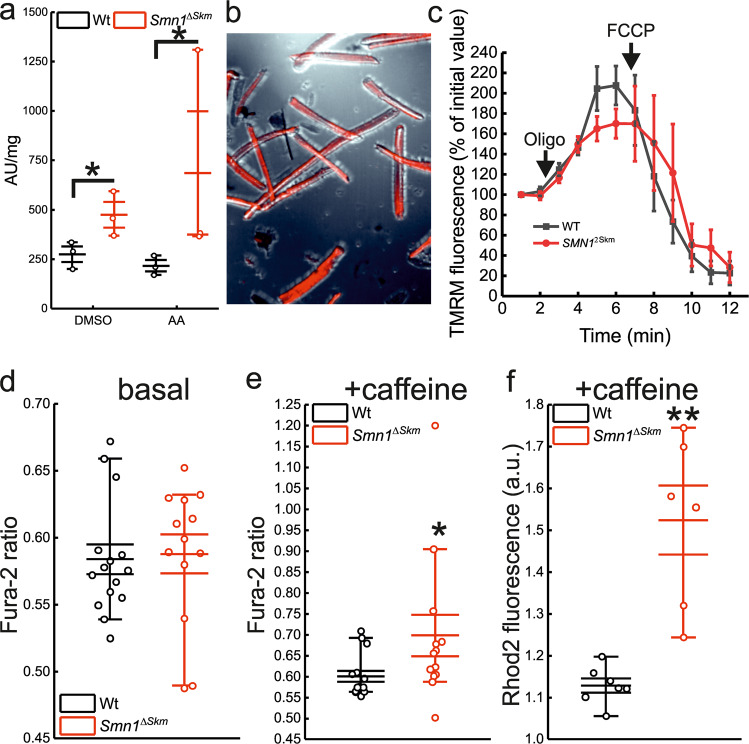
Loss of SMN in skeletal muscle causes oxidative stress and dysregulated Ca^2+^ homeostasis. **a** Box-dots plots of H_2_O_2_ levels in saponin-permeabilized fiber bundles from TA muscles of Wt and *Smn1*^*ΔSkm*^ mice (*n* = 3), without and with addiction of antimycin A (5 μM). The error bars indicate SEM; *p < 0.05. **b** Representative fluorescence image of fibers isolated from *tibialis anterior* (TA) muscle following loading with Tetramethylrhodamine, ethyl ester (TMRM, in Red). **c** The mitochondrial membrane potential of myofibres from Wt and *Smn1*^*ΔSkm*^ mice was measured before and after the addition of 5 μM oligomycin (Frame 2) and 10 μM FCCP (Frame 5-6). Box-dots plots of basal (**d**) and caffeine-induced (**e**) cytoplasmic Ca^2+^ levels in single FDB muscle fibers from mice of the indicated genotypes (*n* = 3). The error bars indicate SEM; *p = 0.02. **f** Box-dots plots of mitochondrial Ca^2+^ levels upon exposure to caffeine in single FDB muscle fibers from mice of the indicated genotypes (*n* = 3). The error bars indicate SEM; **p = 0.003.

Cellular SRC exhaustion can be caused by oxidative stress and/or accumulation of dysfunctional mitochondria, two common facets of neurodegenerative diseases also found in different SMA models [50, 51]. Moreover, the assembly of complex I with complexes III and IV of the mitochondrial respiratory chain to configure I-III- or I-III-IV-containing supercomplexes regulates mitochondrial energy efficiency and reactive oxygen species production. We, therefore, measured H_2_O_2_, the most stable among the ROS, produced by respiring mitochondria in saponin-permeabilized fiber bundles using the fluorescent probe Amplex Red [52]. *Smn1*^*ΔSkm*^ mitochondria released more H_2_O_2_ not only in basal conditions but also upon the supplementation of the complex III inhibitor antimycin A, revealing that these mitochondria are more sensitive to respiratory chain inhibition, as expected [53] (Fig. 5a).

Because mitochondrial ROS can sensitize the mitochondrial permeability transition pore (PTP), leading to its opening, mitochondrial depolarization and swelling, a phenotype that we had observed in our model (Fig. 3), we measured mitochondrial membrane potential in *Smn1*^*ΔSkm*^ fibers. However, the response of *Smn1*^*ΔSkm*^ fibers to the ATP synthase inhibitor oligomycin (a sensitive assay for muscle mitochondrial dysfunction due to PTP opening [54]) was comparable to that of control fibers (Fig. 5b, c). On the other hand, while basal cytosolic Ca^2+^ levels were superimposable in *Smn1*^*ΔSkm*^ and control fibers, caffeine supplementation that induces Ca^2+^ release from sarcoplasmic reticulum led to higher cytosolic and mitochondrial Ca^2+^ transients in *Smn1*^*ΔSkm*^ fibers (Fig. 5d–f). Mitochondrial Ca^2+^ accumulation depends on the Ca^2+^ uptake driving force (membrane potential) as well as on the amount of Ca^2+^ found in microdomains at the ER/SR-mitochondria interface that are essential to activate the mitochondrial Ca^2+^ uniporter [55, 56]. The levels at these microdomains are proportional to the amount of Ca^2+^ released from the ER/SR and can be indirectly inferred from the global cytoplasmic Ca^2+^ levels observed upon activation of the ER/SR Ca^2+^ release pathways. In our conditions, the increased mitochondrial Ca^2+^ uptake observed can be thus explained because of the increased SR Ca^2+^ discharge induced by caffeine and mirrored by the increased cytoplasmic Ca^2+^ levels we measure using Fura 2-AM upon caffeine treatment (Fig. 5e, f). Taken together, these results indicate that *Smn1*^*ΔSkm*^ mitochondria accumulate more Ca^2+^ upon sarcoplasmic reticulum discharge and produce more ROS, potentially sensitizing them to PTP opening and triggering the measured mitochondrial respiratory defect.

### Skeletal muscle SMN loss impairs the clearance of dysfunctional mitochondria

**Fig. 6:**
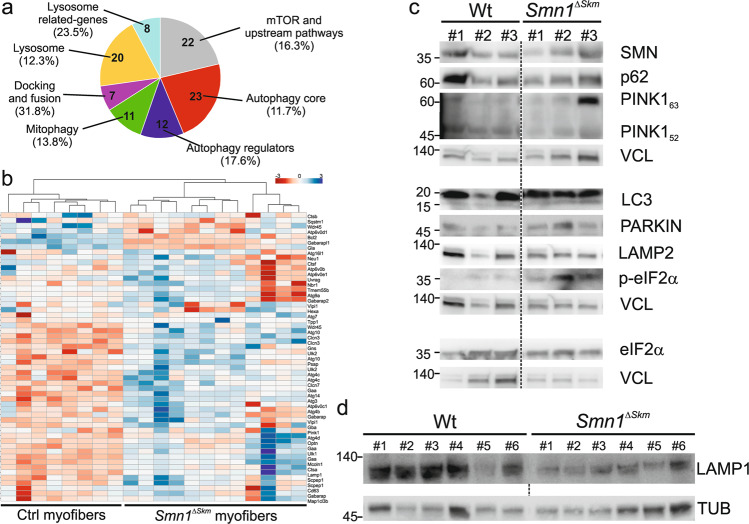
SMN loss in skeletal muscle causes autophagy dysregulation. **a** Pie chart of differentially expressed genes belonging the 6 main groups of the autophagic list [57] in *Smn1*^*ΔSkm*^ myofibers. The percentages indicate the proportion of the genes differentially expressed in *Smn1*^*ΔSkm*^ myofibers over the number of genes belonging to each category as defined in [57]. **b** Heat map of expression levels of genes related to autophagy activation and lysosomal biogenesis. Each column corresponds to a single myofiber of the indicated genotype. The dendrogram cluster myofibers with similar transcriptional signature. **c**, **d** Equal amounts of *gastrocnemius* muscle lysates (20 µg) were separated by SDS-PAGE and immunoblotted using the indicated antibodies. Each lane corresponds to an individual mouse of the indicated genotypes. Densitometry analysis yielded the following values: SMN: 1.16 ± 0.14 in Wt (*n* = 4) and 0.77 ± 0.04 in *Smn1*^*ΔSkm*^ (*n* = 5), *p* = 0.02; LC3II: 0.04 ± 0.01 in Wt (n = 4) and 0.11 ± 0.02 in *Smn1*^*ΔSkm*^ (*n* = 5), *p* = 0.03; PINK: 0.79 ± 0.14 in Wt (*n* = 4) and 1.6 ± 0.29 in *Smn1*^*ΔSkm*^ (*n* = 4), *p* = 0.04; PARKIN: 0.14 ± 0.02 in Wt (*n* = 4) and 0.8 ± 0.17 in *Smn1*^*ΔSkm*^ (*n* = 4), *p* = 0.01 p-eIF2α/ eIF2αː 0.19 ± 0.02 in Wt (*n* = 4) and 0.4 ± 0.05 in *Smn1*^*ΔSkm*^ (*n* = 4), *p* = 0.01; LAMP2 Wt 0.79 ± 0.11 in Wt (*n* = 5) and 0.48 ± 0.03 in *Smn1*^*ΔSkm*^ (*n* = 7), *p* = 0.013; and LAMP1: 0.75 ± 0.14 in Wt (*n* = 6) and 0.37 ± 0.06 in *Smn1*^*ΔSkm*^ (*n* = 6), *p* = 0.037.

Dysfunctional, ROS-generating mitochondria are normally eliminated by mitophagy that, in turn, serves to reduce oxidative stress. Since we observed that damaged mitochondria accumulate in *Smn1*^*ΔSkm*^ muscles, we hypothesized that these mitochondria might not be removed by mitophagy. We went back to our transcriptomic data to verify whether mitochondrial abnormalities were accompanied by changes in autophagy–lysosome degradation systems. Using an innovative transcriptional toolbox for deducing autophagy status and lysosomal biogenesis [57], we found that about 15% of the 604 genes composing the entire autophagic list are differentially expressed in the myofibers of *Smn1*^*ΔSkm*^ mice (Fig. 6a, Supplementary Table 3). Restricted lists of genes identified as the “induction list” of autophagy-related transcripts and lysosomal biogenesis-associated genes [57] were found mostly reduced in the myofibers of *Smn1*^*ΔSkm*^ mice (Fig. 6b). Notably, protein levels of PINK, PARKIN and LC3II were found increased in muscles isolated from *Smn1*^*ΔSkm*^ mice compared to Wt controls. Phosphorylated eIF2α that plays a central role in autophagy induction [58], was found significantly higher too (Fig. 6c). However, despite the damaged *Smn1*^*ΔSkm*^ mitochondria can be marked for degradation, their degradation process appears to be uncompleted. In fact, levels of the proteins LAMP1 and LAMP2, essential for maintaining lysosome level, were reduced, suggesting autolysosomes dysfunction (Fig. 6c, d).

**Fig. 7:**
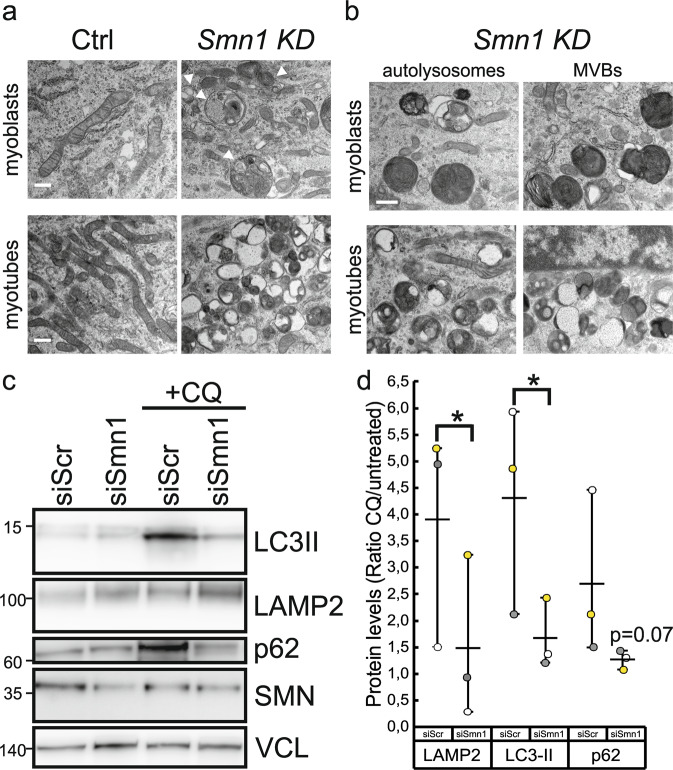
*Smn1* deficient muscle cells accumulate autolysosomes and multivescicular bodies (MVBs). **a** Representative electron microscopy images showing increased number of autolysosomes and MVBs (white arrows) upon Smn1 knockdown in C2C12 myoblasts and differentiated myotubes (*n* = 3 independent experiments). Scale bar: 10 μm. **b** Representative high magnification of *Smn1* deficient muscle cells. Scale bar: 500 nm. **c** Equal amounts of siScr and siSmn1 transfected C2C12 lysates (20 µg) were separated by SDS-PAGE and immunoblotted using the indicated antibodies. Where indicated the cells were treated with chloroquine (CQ) before collection. **d** Dots plot of the differences in the amount of LAMP2, LC3-II and p62 between samples in the presence and absence of CQ as a measure of the autophagic flux in siScr and siSmn1 transfected cells (*n* = 3 paired biological replicates represented by different colors). *p* value was calculated using ratio paired t test one tail statistical analysis; LAMP2 and LC3-II, *p = 0.04; p62, *p* = 0.07.

As LC3-II is both produced and degraded during autophagy, to check whether autolysosomes accumulation is due to autophagy induction or rather a block in downstream steps we evaluated LC3II protein level changes in the presence and absence of the autophagy inhibitor cloroquine. We used C2C12 cells, a well-established model of muscle cells. First, we showed that the silencing of *Smn1* in proliferating myoblasts as well as myotubes causes the accumulation of autolysosomes and multivescicular bodies (MVBs) (Fig. 7a, b), similarly to what was observed in vivo (Fig. 3a, b). In order to understand in which direction autophagy was altered, we inspected autophagic flux in control and *Smn1* deficient myoblasts by using chloroquine. The difference in the levels of key autophagy-lysosomal markers in the presence and absence of chloroquine is larger in control compared to *Smn1*-silenced cells indicating that autophagic flux is reduced upon *Smn1* deficiency (Fig. 7c, d). Overall, these data support that dysfunctional mitochondrial clearance in myofibers of *Smn1*^*ΔSkm*^ mice is impaired because of altered autophagy.

### AFS cells transplantation in *Smn1*^*ΔSkm*^ mice corrects expression of mitochondrial transcripts and restores mitochondrial morphology

**Fig. 8:**
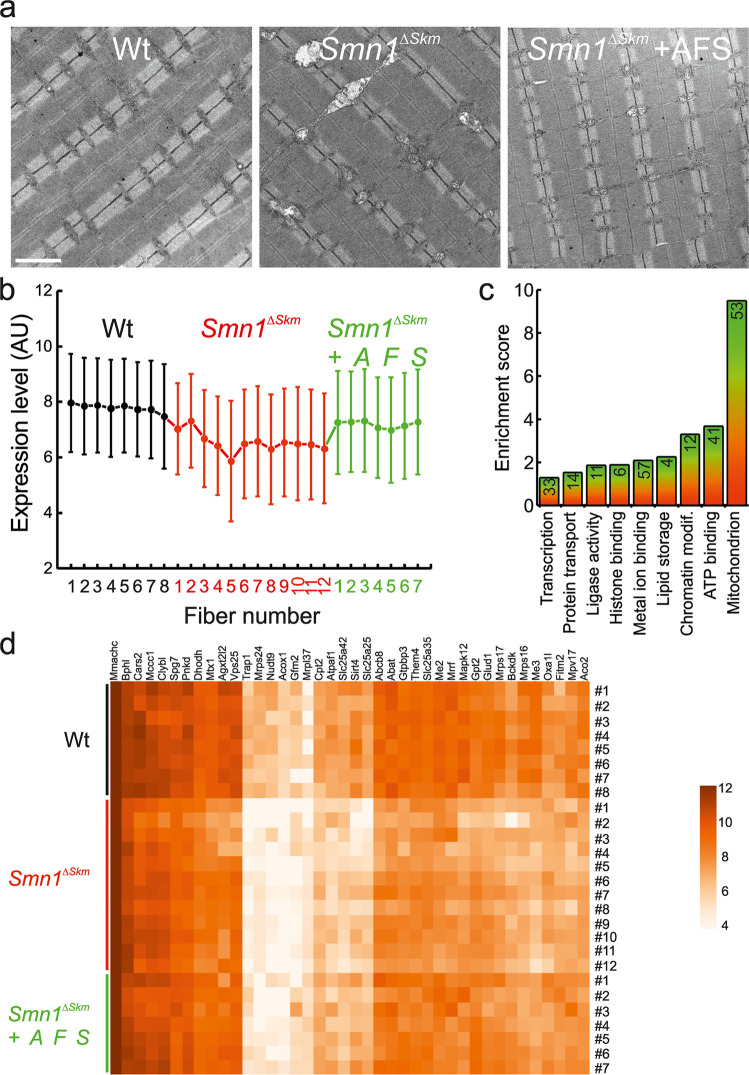
AFS transplantation in *Smn1*^*ΔSkm*^ mice rescues mitochondrial alterations. **a** Representative electron micrographs of mitochondria from TA muscles of Wt and untreated or AFS-treated *Smn1*^*ΔSkm*^ mice. Scale bar: 2 μm. **b** Expression pattern of genes obtained by Pavlidis Template Matching algorithm that match the template expression profile of high expression in Wt myofibers, low in the *Smn1*^*ΔSkm*^ myofibers, and high in the *Smn1*^*ΔSkm*^ + AFS myofibers (threshold R of 0.7). **c** Gene ontology analysis of the genes with the expression profile in **b**. Enrichment score by DAVID was used to rank the annotation groups. The numbers inside bars correspond to the number of genes for each cluster. **d** Heat-map of the genes with the expression profile pattern in **b**) distinguishes the signatures of myofibers isolated from *tibialis anterior* muscles of Wt and untreated or AFS-treated *Smn1*^*ΔSkm*^ mice.

Tail vein transplanted mouse AFS cells integrate into the muscle stem cell compartment, improving muscle strength and survival rate of *Smn1*^*ΔSkm*^ mice [25]. We therefore wished to evaluate whether mouse AFS cells transplantation could also lead to mitochondrial amelioration in *Smn1*^*ΔSkm*^ muscles. We tail vein injected GFP + AFS cells and first evaluated mitochondrial ultrastructure in TA muscle. When *Smn1*^*ΔSkm*^ mice were AFS-treated, mitochondrial morphological abnormalities were largely corrected (Fig. 8a). With the support of these results, we turned to our transcriptional analysis approach to investigate whether AFS cell transplantation could also correct the changes in gene expression observed in *Smn1*^*ΔSkm*^ mice. We performed a transcriptome analysis of five single fibers isolated from TA muscles of three mice sacrificed four weeks after AFS cell transplantation. By using the Pavlidis Template Matching [31] implemented in MultiExperiment Viewer [59] we marked the genes that were under-expressed in *Smn1*^*ΔSkm*^ myofibers and returned to control expression levels after AFS cells treatment. We set as template high expression for control untreated and AFS-treated myofibers, and low expression for *Smn1*^*ΔSkm*^ myofibers with a threshold R of 0.7. The Algorithm identified 285 genes (Fig. 8b, the complete list can be found in Supplementary Table 4). The functional annotation of this set of genes showed that it was enriched for transcripts involved in mitochondrial function, especially protein translation, and in lipid metabolic processes (Fig. 8c, d). Altogether, these data suggest that AFS cell transplantation can restore not only muscle function, but also mitochondrial gene expression and mitochondrial morphology.

## Discussion

The approved therapies for SMA aim at rescuing the loss of the survival motor neuron (SMN1) protein almost exclusively in motor neuron populations. However, other cell types and tissues where this gene is expressed, are intrinsically affected by depletion of SMN1 and this feature significantly contributed to the failure of the current molecular treatments to cure SMA. As skeletal muscle is one of the most vulnerable tissues to SMN1 insufficiency, revealing muscle-autonomous molecular mechanisms in SMA disease is crucial for developing therapies that prevent the loss of skeletal muscle and that can be combined with the restoration of SMN in motor neurons. Here we applied high resolution transcriptional analyses on isolated myofibers to unmask the transcriptional profile of individual SMA affected muscle fibers. We used an extensively characterized mouse model in which the murine Smn exon 7 is flanked by two LoxP sequences and deletion is occurring only in skeletal myofibers by placing the Cre recombinase under the control of the human α‐skeletal actin gene promoter. Our single myofiber analysis revealed myofiber-specific gene expression patterns that are altered upon *Smn1* loss. Unsurprisingly, given the well-known nuclear role of SMN, more than 200 genes associated with transcription were found differentially expressed in *Smn1*^*ΔSkm*^ myofibers compared with controls. Moreover, 73 translation-related transcripts were found downregulated, confirming the role of SMN in RNA synthesis and metabolism [60]. Interestingly our gene ontology analysis pointed out to a signature of mitochondrial dysfunction. Therefore, we inspected mitochondrial morphology and function in muscles from *Smn1*^*ΔSkm*^ mice. Electron microscopy analysis showed mitochondrial vacuolar degeneration: several grades of degeneration were recognized, from concentric, onion-like arrangement of mitochondrial cristae to mitochondrial swelling. However, several unaltered mitochondria were also observed, suggesting a possible compensatory effect of increased biogenesis, as confirmed by the higher mitochondrial proteins levels found in *Smn1*^*ΔSkm*^ muscle This could be a consequence of the continuous regeneration of the muscle compartment by muscle stem cells [61] which could lead to the retrieval of these “normal” mitochondria. While rates of oxygen consumption were comparable in Wt and *Smn1*^*ΔSkm*^ muscles, *Smn1*^*ΔSkm*^ muscles showed strongly reduced mitochondrial spare respiratory capacity compared to Wt. The decline in spare reserve capacity was found associated with a reduction in the activity of the complex IV-formed respiratory supercomplexes that provide higher efficiency during ETC minimizing the generation of ROS [62]. This could result from defects in the assembly of COX2 since *Cox18* and *Tmem177* were found strongly reduced in *Smn1*^*ΔSkm*^ myofibers. Accordingly, despite no overall decrease amount of complex I, its enzymatic activity that is dependent on the presence of complex IV [63], was strongly reduced in *Smn1*^*ΔSkm*^ muscle compared to Wt. Accordingly, as complex I is one of the major site for ROS production, high levels of ROS and Ca^2+^ uptake characterized the *Smn1*^*ΔSkm*^ muscle fibers mitochondria, pointing to the possibility that opening of the PTP was favored by SMN loss and potentially explaining the extensive mitochondrial swelling observed in the *Smn1*^*ΔSkm*^ muscles. However, we failed to record signs of PTP opening in experiments of real time membrane potential measurements in response to oligomycin. Whether this failure was due to experimental limitations (i.e., fiber contraction during oligomycin treatment) or truly to the lack of PTP opening remains to be elucidated.

Often, the accumulation of morphologically altered mitochondria results from a failure in mitophagy. Our transcriptomic data pointed to this direction: *Bnip3* and *Fundc1*, the best known membrane receptors linked to hypoxia-induced mitophagy, were significantly downregulated in *Smn1*^*ΔSkm*^ myofibers, while *Park2* was increased, probably as a compensatory mechanism for the inefficient removal of damaged mitochondria. Increased protein levels of LC3II and increased phosphorylation of the ER stress protein eIF2α corroborated our hypothesis. Efficient autophagy requires the simultaneous increase in lysosomal activity, to ensure the degradation of autophagic substrates by mobilizing a variety of proteins, lipids and intracellular membranes of different organelles [64, 65]. Notably, the lysosomal-associated membrane protein LAMP was reduced in muscle of *Smn1*^*ΔSkm*^ mice potentially explaining the lysosomal facet of mitophagy failure. To understand whether altered autophagy was responsible for the accumulation of damaged mitochondria in the *Smn1* deficient muscle cells, we assessed the autophagic flux. Our data show that *Smn1* deficiency causes autophagy/mitophagy alterations and finally defective mitochondrial clearance.

Interestingly, when we infused *Smn1*^*ΔSkm*^ mice with AFS cells that correct their muscle phenotype, ultrastructural alterations in mitochondria were corrected. We investigated the reprogramming of the transcriptome in ASF-transplanted myofibers and we found that expression of several mitochondrial genes that were downregulated upon SMN loss returned to Wt levels.

Our results corroborate the concept that mitochondria may represent a relevant target for a combinatorial therapy in SMA disease. Indeed, the PTP modulator Olesoxime, a promising drug that preserves mitochondrial homeostasis, motoneuron integrity in SMA and increases motor function in SMA patients [66, 67] entered phase III clinical trial. This was then stopped because of a commercial decision by the owner of the drug. However, given the mounting evidence for a role of mitochondria in SMA pathophysiology, this cancellation could be envisaged as premature from a scientific point of view. Novel mitochondrially-targeted therapies are needed especially for adults with Type 2/3 SMA since the current gene treatments need to be started earlier, before motor symptoms develop, to achieve the highest probability of success.

AFS cells transplantation can be envisaged as a biological treatment to restore mitochondrial homeostasis in SMA. Our previous data show that AFS GFP + cells integrate in the muscle stem cell compartment with long-term potential for muscle regeneration in HSA-Cre, SmnF7/F7 mutant mice, being able to become muscle cells after systemic injection [25]. These experiments tend to support a model whereby the correction is due to the replenishment of the muscle stem cell niche with functioning AFS cells. At the same time, it is possible that mitochondria from the AFS cells are transferred to Smn1-/- muscle, thereby contributing to the amelioration of the phenotype by a multipronged mechanism that comprises the repopulation of the muscle stem cell compartment with healthy AFS derived stem cells, the correction of the Smn1-/- stem cells mitochondrial population by transfer of mitochondria from the AFS cells, and even the correction of the mature muscle mitochondria by AFS derived mitochondria.

Regarding the therapeutic perspective, human AFS cells from the second and third trimesters of gestation display a low immunogenic profile [68] and appear safe [69]. Moreover, given their stem-like properties a very low number of AFS cells can be transplanted to achieve clinical efficacy. This was the case in our mouse model and a similar scenario can be envisioned in SMA pediatric patients where AFS transplantation can be used to restore skeletal muscle mitochondrial integrity, in combination with the approved gene therapies.

## Supplementary information

Supplemental Figure S1

Original western blots

Supplemental Table 1

Supplemental Table 2

Supplemental Table 3

Supplemental Table 4

Reproducibility Checklist
