# Supplementary material for: Dysfunctional mitochondria accumulate in a skeletal muscle knockout model of Smn1, the causal gene of spinal muscular atrophy
Source: Cell Death Dis. 2023 Feb 27;14(2):162. doi: 10.1038/s41419-023-05573-x (PMC9971247; doi:10.1038/s41419-023-05573-x)
Supplement: Supplementary file 1 — Supplemental Figure S1 [file 41419_2023_5573_MOESM1_ESM.docx]

**Supplemental Figure S1** *related to Figure 4*


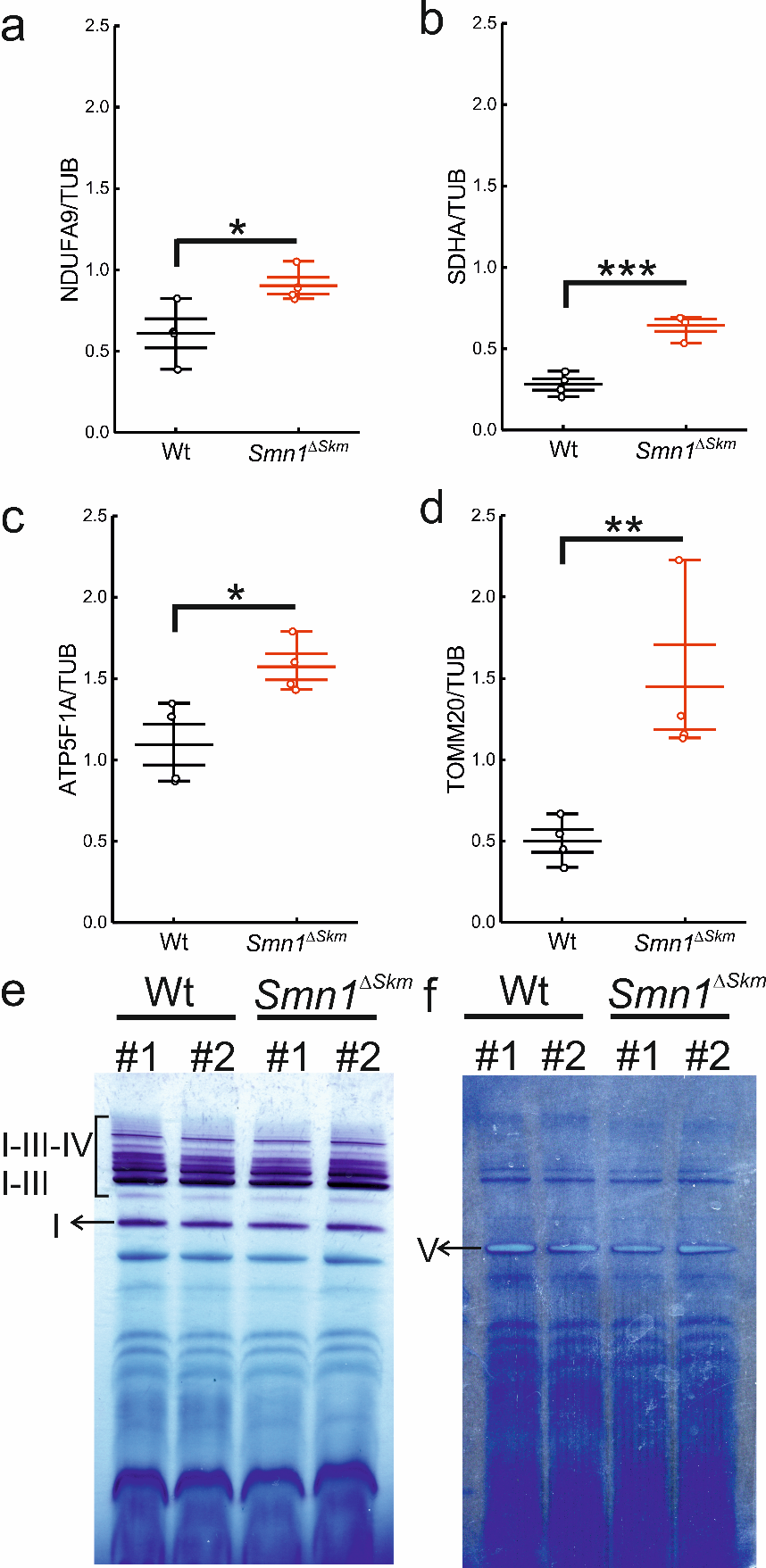


**Supplemental Figure S1. SMN1 deficiency causes changes in the expression of key mitochondrial proteins.** a-d) Box-dots plots of the densitometry analyses for NDUFA9 (a), SDHA (b), ATP5F1A (c) and TOMM20 (d).TUB was used as loading control. The error bars indicate SEM; *p<0.05; **p<0.01; ***p<0.001. e) CI-specific BNGE in-gel activity. No significant difference was detected in the staining of the bands corresponding to CI-holoenzyme and CI-containing sc. d) CV-specific BNGE in-gel activity. No significant difference was detected in the staining of the band corresponding to CV-holoenzyme.
