# Supplementary figures and images for: Dysfunctional mitochondria accumulate in a skeletal muscle knockout model of Smn1, the causal gene of spinal muscular atrophy
Source: Cell Death Dis. 2023 Feb 27;14(2):162. doi: 10.1038/s41419-023-05573-x (PMC9971247; doi:10.1038/s41419-023-05573-x)

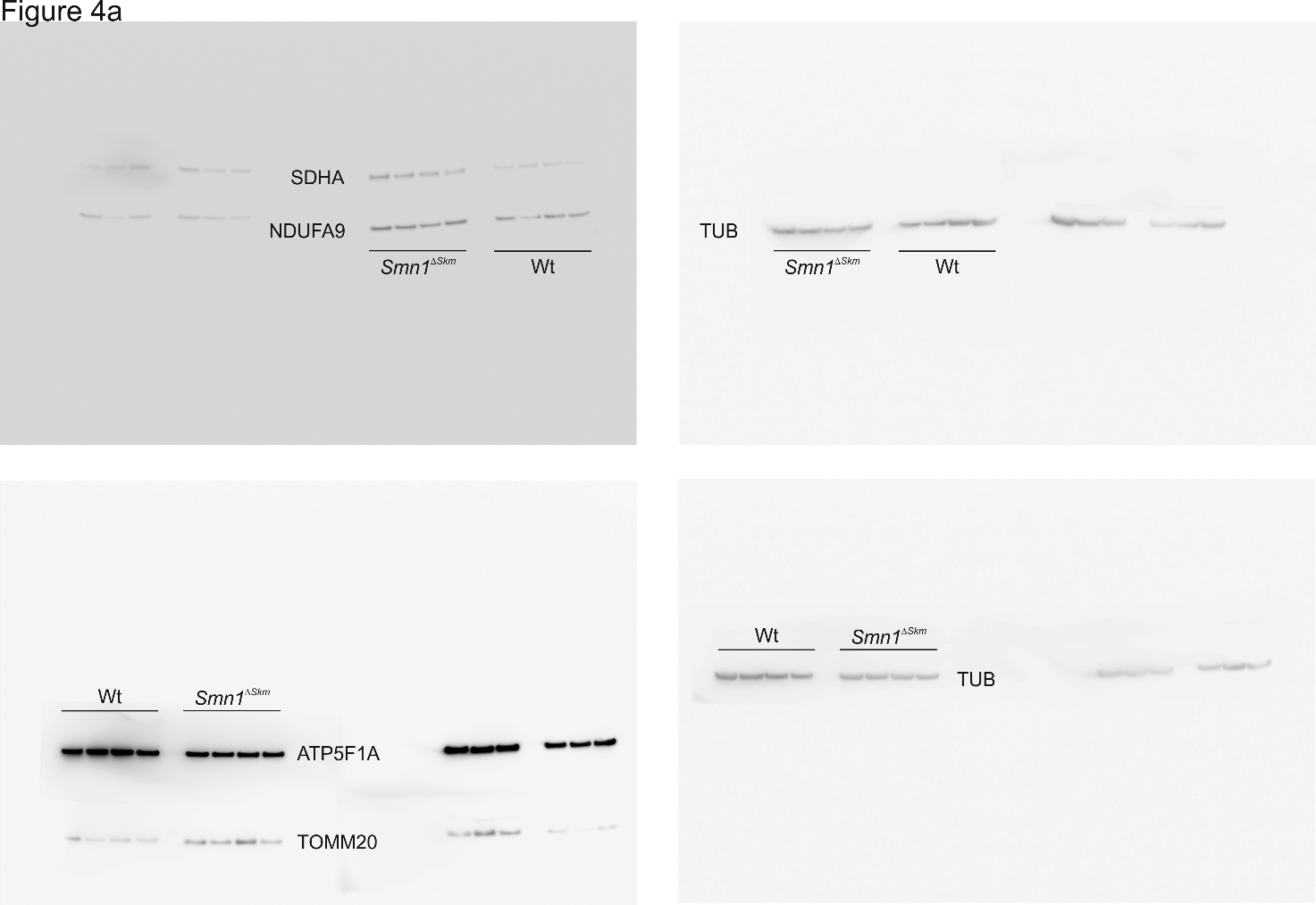


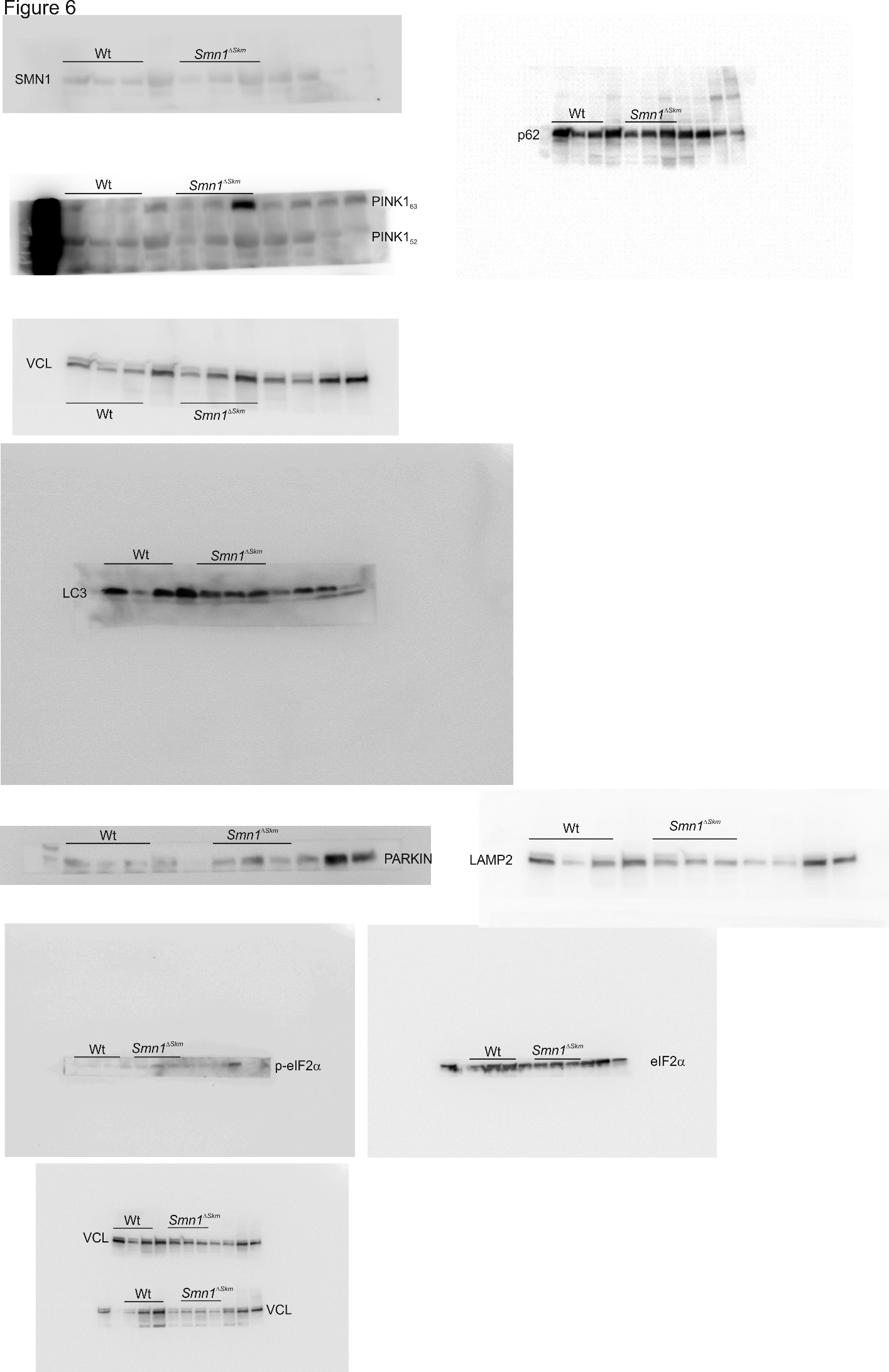


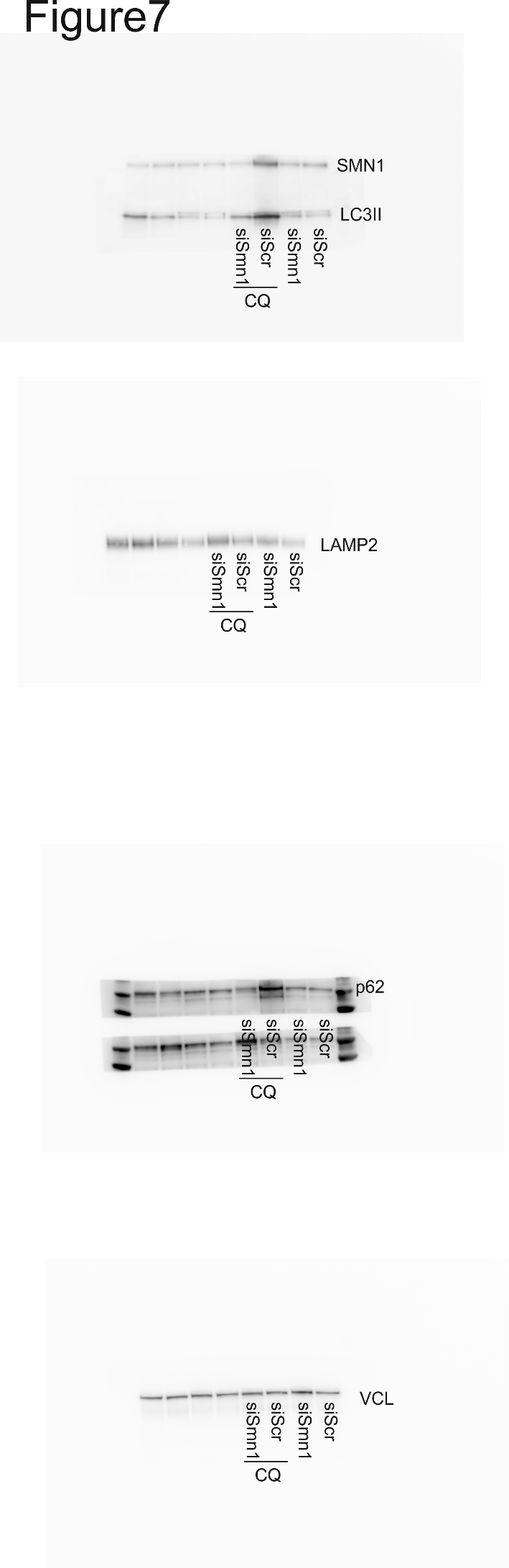

Supplement: Supplementary file 2 — Original western blots [file 41419_2023_5573_MOESM2_ESM.docx]
